# Supplementary material for: Early Low-Fluence Red Light or Darkness Modulates the Shoot Regeneration Capacity of Excised Arabidopsis Roots
Source: Plants (Basel). 2020 Oct 16;9(10):1378. doi: 10.3390/plants9101378 (PMC7602781; doi:10.3390/plants9101378)
Supplement: Supplementary file 1 [file plants-09-01378-s001.pdf]

plants

MDPI

Supplemental Fig. 1

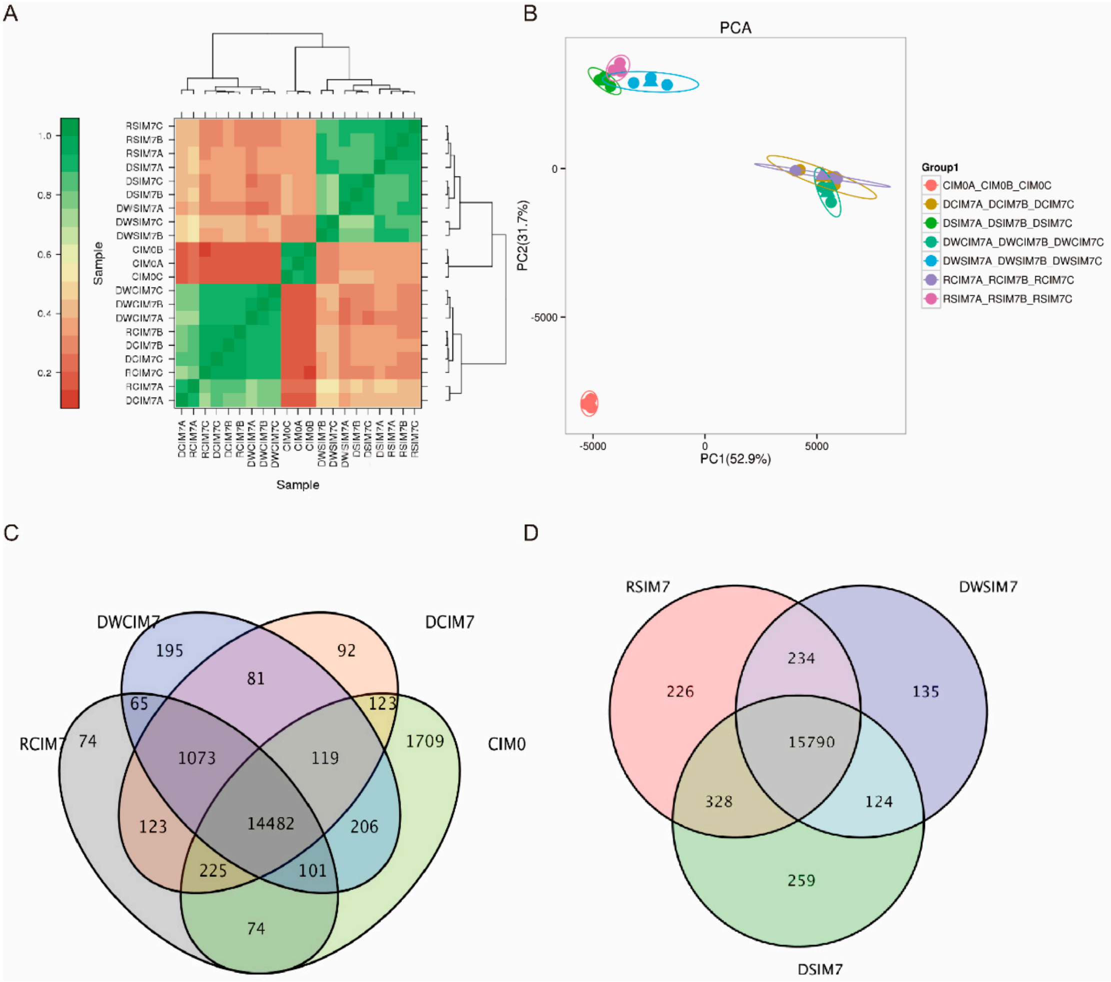

Figure S1. Overall qualitative analysis of the transcriptomics data. **A** Pearson's correlation coefficients among the three samples (CIM0, RCIM7, DCIM7, DWCIM7, RSIM7, DSIM7 and DWSIM7). B PCA analysis of the seven samples. the x-axis represents the first principal component (PC1) and the y-axis represents the second principal component (PC2). **C** Co-expression of Venn diagrams in the group 2 (DWCIM7, DCIM7, RCIM7). **D** Co-expression of Venn diagrams in the group 3 (DWSIM7, DSIM7, RSIM7). The Numbers in a Venn diagram represent the number of specific or common genes expressed. The overlapping region represents the number of genes expressed in different samples, while the non-overlapping region represents the number of genes expressed in different samples. CIM0 (CIM 0 d); RCIM7 (24hR-W treatment, CIM 7 d); DCIM7 (24hD-W treatment, CIM 7 d) ; DWCIM7 (D-W treatment, CIM 7 d); RSIM7 (24hR-W treatment, SIM 7 d); DSIM7 (24hD-W treatment, SIM 7 d) and DWSIM7 (D-W treatment, SIM 7 d); D-W (the control treatment); 24hD-W, early 24 hours dark and then shifting to 6 days' white light in CIM followed by white light throughout SIM; 24hR-W, early 24 hours red light shifting to 6 days' white light in CIM, followed by white light treatment in SIM; CIM, callus induction medium; SIM, shoot induction medium; PCA, principal component analysis.

plants

Supplemental Fig. 2

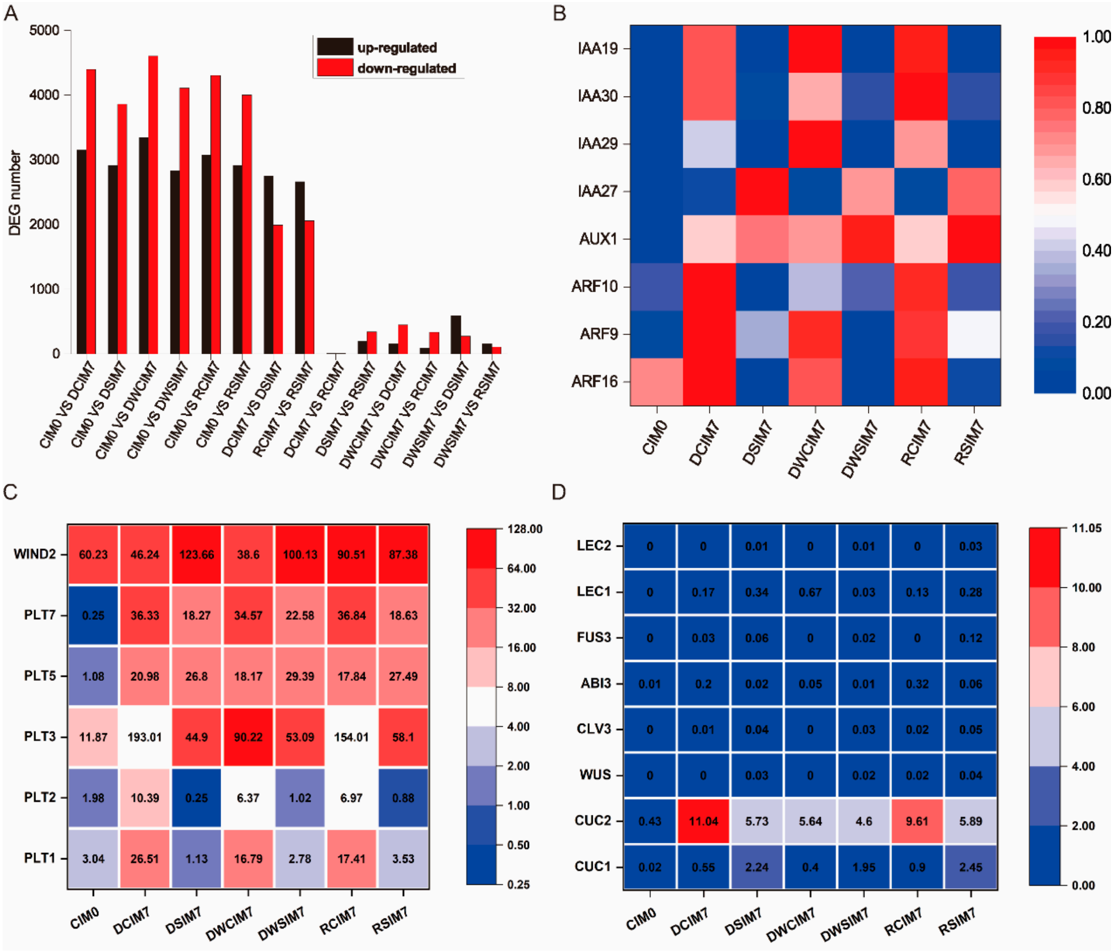

Figure S2. Number of DEGs and Heat maps of auxin responsive and meristem development genes at comparison of different processing combinations. **A** Number of DEGs at comparison of different processing combinations. **B** Heat map of auxin response gene expression at early stage. **C** Heat map of RAM genes expression at early stage. **D** Heat map of SAM genes expression at early stage. CIM0 (CIM 0 d); RCIM7 (24hR-W treatment, CIM 7 d); DCIM7 (24hD-W treatment, CIM 7 d); DWCIM7 (D-W treatment, CIM 7 d); RSIM7 (24hR-W treatment, SIM 7 d); DSIM7 (24hD-W treatment, SIM 7 d) and DWSIM7 (D-W treatment, SIM 7 d); D-W (the control treatment); 24hD-W, early 24 hours dark and then shifting to 6 days' white light in CIM followed by white light throughout SIM; 24hR-W, early 24 hours red light shifting to 6 days' white light in CIM, followed by white light treatment in SIM; CIM, callus induction medium; SIM, shoot induction medium.

Supplemental Fig. 3

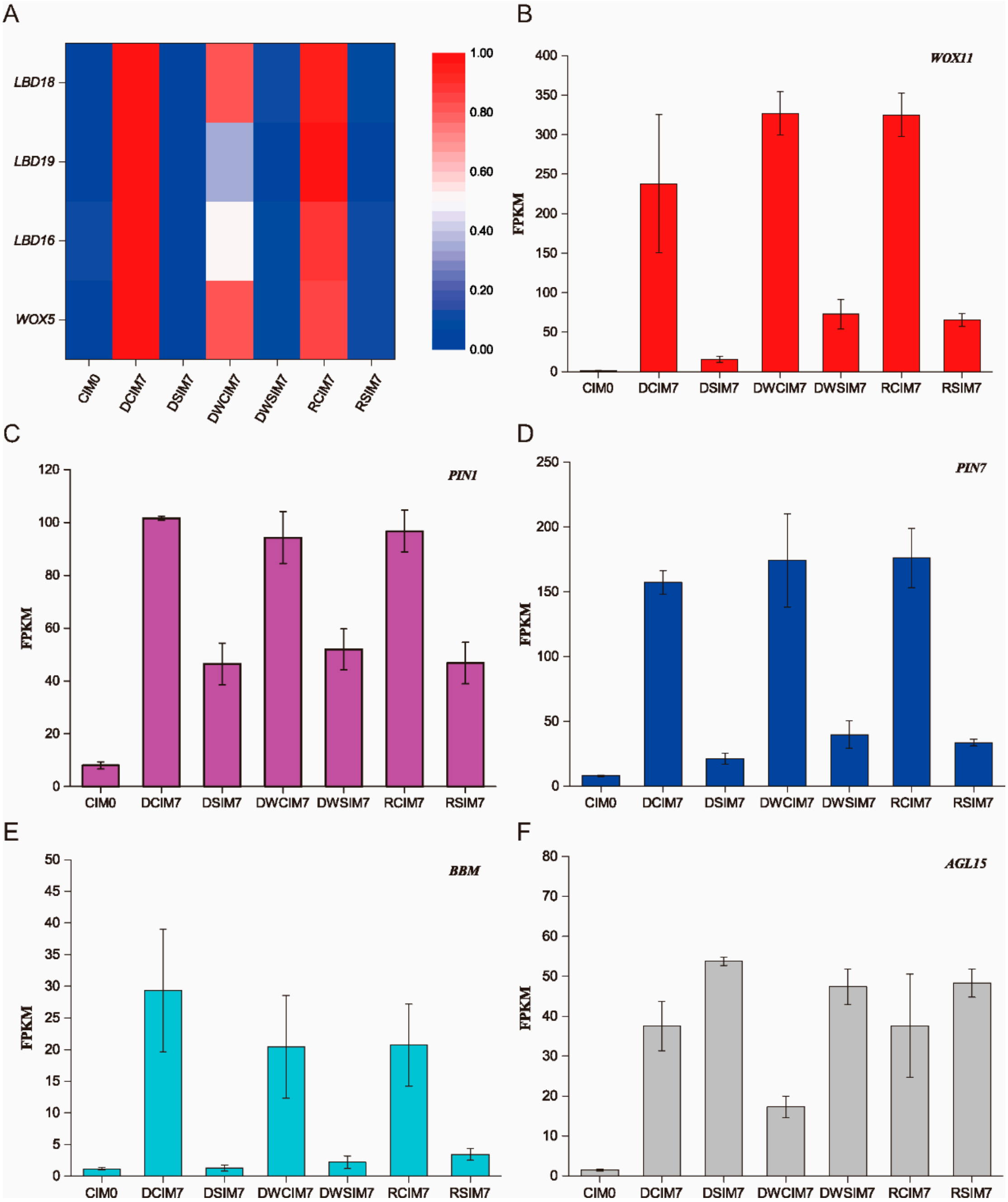

Figure S3. Transcriptome analysis of marker genes expression patterns in the CIM and SIM stages. **A** Heat map showing the callus-induced marker genes expression patterns of *LBD18*, *LBD16*, *LBD19* and *WOX5* in in the CIM and SIM stages under light under the early low-fluence red light or darkness. **B** Expression patterns of the *WOX11* of the *Arabidopsis thaliana* WUSCHEL-related homeobox gene family member under different treatments. c-d Expression patterns of the *PIN1*(**C**) and *PIN7* (**D**) of the *Arabidopsis thaliana* WUSCHEL-related homeobox

gene family member under different treatments. **E** Expression patterns of the BBM under different treatments. **F** Expression patterns of the *AGL15* of the member of the MADS domain family of regulatory factors under different treatments. CIM0 (CIM 0 d); RCIM7 (24hR-W treatment, CIM 7 d); DCIM7 (24hD-W treatment, CIM 7 d); DWCIM7 (D-W treatment, CIM 7 d); RSIM7 (24hR-W treatment, SIM 7 d); DSIM7 (24hD-W treatment, SIM 7 d) and DWSIM7 (D-W treatment, SIM 7 d); D-W (the control treatment); 24hD-W, early 24 hours dark and then shifting to 6 days' white light in CIM followed by white light throughout SIM; 24hR-W, early 24 hours red light shifting to 6 days' white light in CIM, followed by white light treatment in SIM; CIM, callus induction medium; SIM, shoot induction medium; LBD, LOB domain-containing protein.

Supplemental Fig. 4

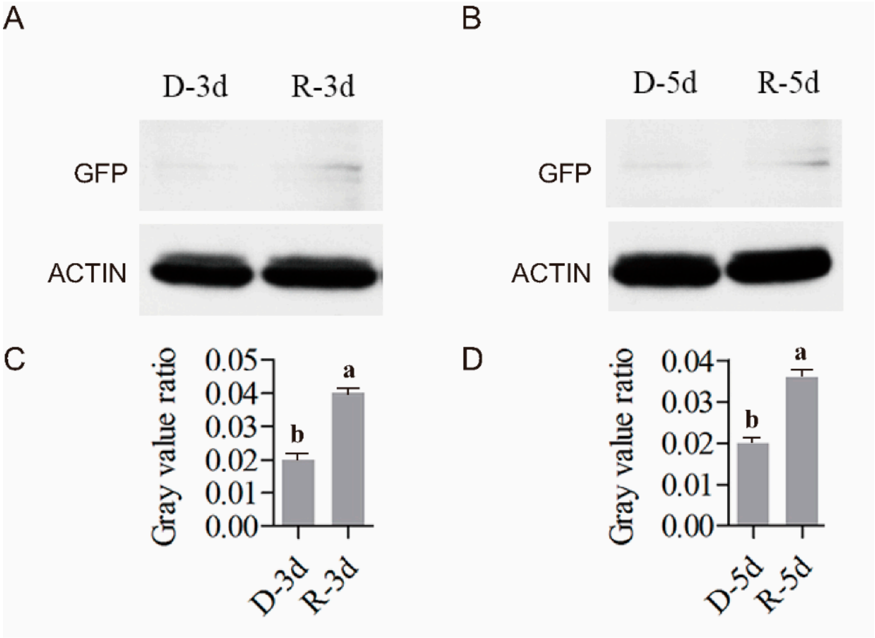

Figure S4. A western blot shows the kinetic of auxin accumulation in D-3d, R-3d, D-5d and R-5d. **A** Western blot shows auxin accumulation in D-3d and R-3d. **B** A western blot shows auxin accumulation in D-5d and R-5d. **C** A gray value ratio shows auxin accumulation in D-3d and R-3d. **D** A gray value ratio shows auxin accumulation in D-5d and R-5d. The ACTIN protein was used as an internal control. GFP-fusion transgenic plants were used for WB analysis with anti-GFP antibodies. D-3d, dark treatment for 3 days in the CIM; R-3d, 24hR-W treatment, CIM 3 d; D-5d, dark treatment for 3 days in the CIM; R-5d, 24hR-W treatment, CIM 5 d; 24hR-W, early 24 hours red light shifting to 6 days' white light in CIM, followed by white light treatment in SIM; CIM, callus induction medium; SIM, shoot induction medium; WB, western blot. The least significant difference method (LSD) was used for significance test ($p < 0.05$); Different lowercase letters represent statistical differences in pairwise comparisons between LSD test groups ($p < 0.05$).

Supplemental Fig. 5

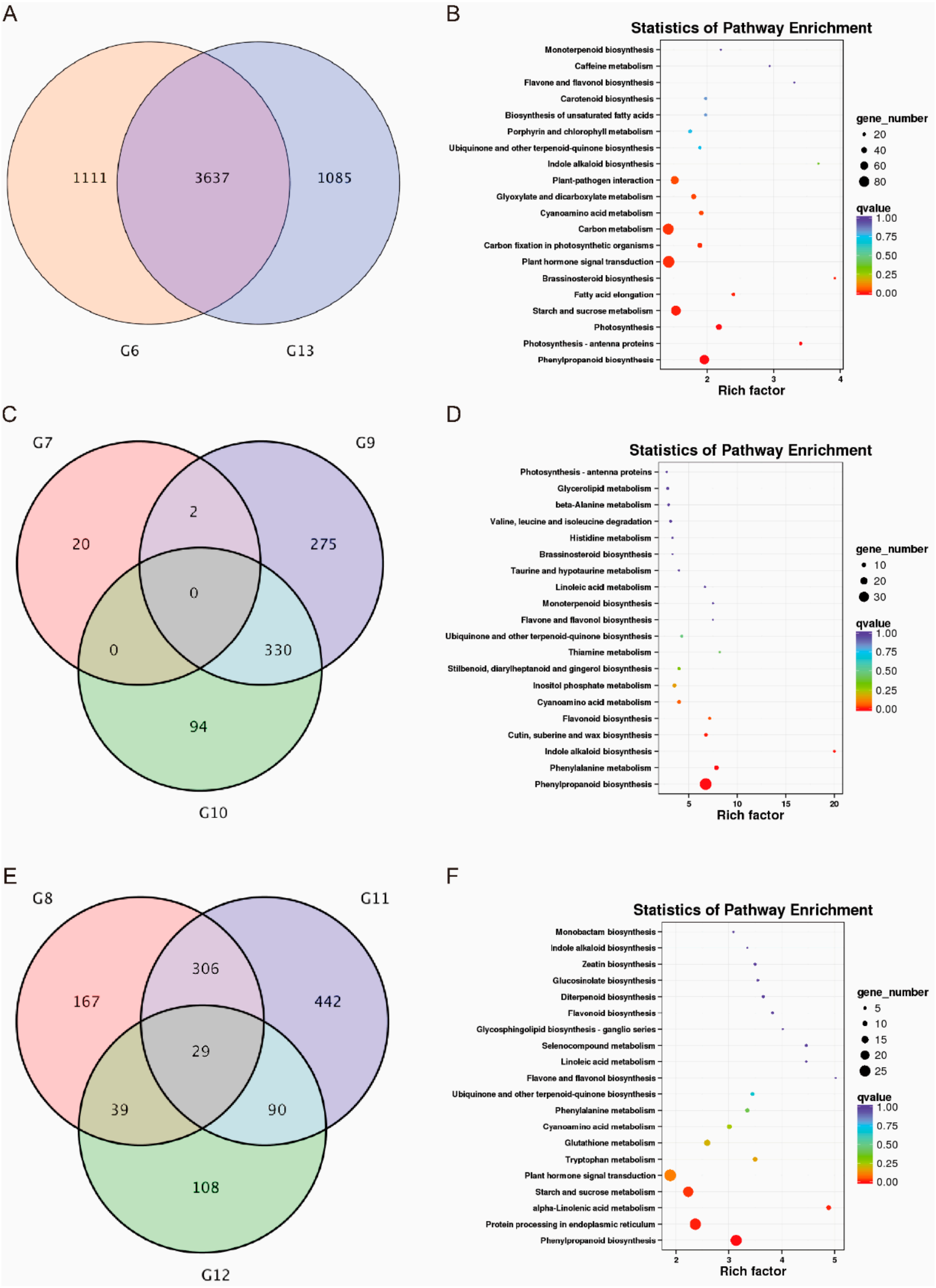

Figure S5. Differential genes commonly and KEGG pathway enrichment analysis of DEGs. **A** Venn diagrams showing the distribution of DEGs in G6 vs. G13 (G6, DCIM7 vs. DSIM7, G13, RCIM7 vs. RSIM7); **B** Pathway enrichment in G6 vs. G13; c Venn diagrams showing the distribution of DEGs in G7 vs. G9 vs. G10 (G7, DCIM7 vs. RCIM7, G9, DWCIM7 vs. DCIM7, G10, DWCIM7 vs. RCIM7); **D** Pathway enrichment in G7 vs. G9 vs. G10; **E** Venn diagrams showing the distribution of DEGs in G8 vs. G11 vs. G12 (G8, DSIM7 vs. RSIM7, G11, DWSIM7

vs. DSIM7, G12, DWSIM7 vs. RSIM7); **F** Pathway enrichment in G8 vs. G11 vs. G12. The x-axis represents the enrichment factor, while the y-axis represents the enrichment pathway. The size of q-value is represented by the color of the dot. The smaller the q-value is, the closer the color is to red. The number of DEGs contained in each function is represented by the size of the dot. The statistical analysis of the pathway enrichment was performed using Fisher's exact test. DEGs, differentially expressed genes; RCIM7 (24hR-W treatment, CIM 7 d); DCIM7 (24hD-W treatment, CIM 7 d) ; DWCIM7 (D-W treatment, CIM 7 d); RSIM7 (24hR-W treatment, SIM 7 d); DSIM7 (24hD-W treatment, SIM 7 d) and DWSIM7 (D-W treatment, SIM 7 d); D-W (the control treatment); 24hD-W, early 24 hours dark and then shifting to 6 days' white light in CIM followed by white light throughout SIM; 24hR-W, early 24 hours red light shifting to 6 days' white light in CIM, followed by white light treatment in SIM; CIM, callus induction medium; SIM, shoot induction medium; KEGG, Kyoto Encyclopedia of Genes and Genomes.

Supplemental Fig. 6

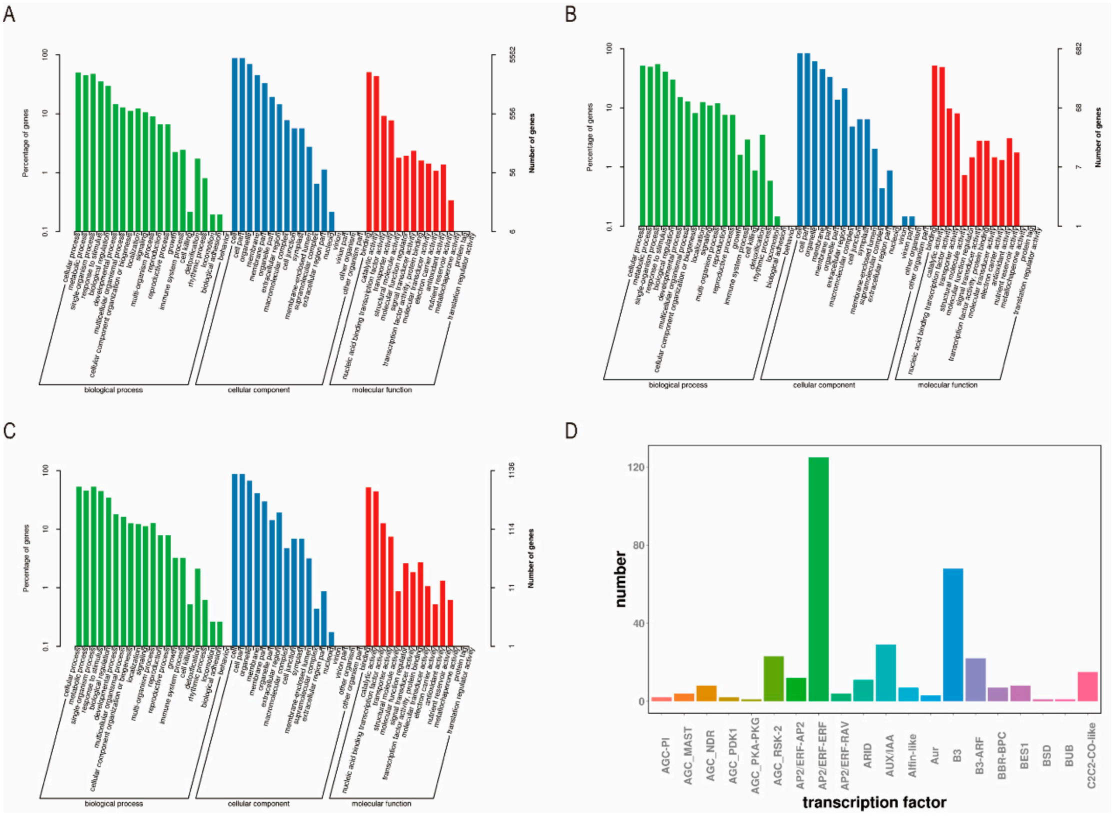

Figure S6. Gene Ontology (GO) annotation and transcription factor prediction in the three stages. **A** GO annotation in the transitional stage (DCIM7 vs. DSIM7, RCIM7 vs. RSIM7); **B** GO annotation in the dedifferentiation stage (DWCIM7 vs. DCIM7, DCIM7 vs. RCIM7 , DWCIM7 vs. RCIM7); c GO annotation in the primary regeneration shoot stage (DWSIM7 vs. DSIM7, DWSIM7 vs. RSIM7, DSIM7 vs. RSIM7); **D** Transcription factor prediction in the three stages. RCIM7 (24hR-W treatment, CIM 7 d); DCIM7 (24hD-W treatment, CIM 7 d) ; DWCIM7 (D-W treatment, CIM 7 d); RSIM7 (24hR-W treatment, SIM 7 d); DSIM7 (24hD-W treatment, SIM 7 d) and DWSIM7 (D-W treatment, SIM 7 d); D-W (the control treatment); 24hD-W, early 24 hours dark and then shifting to 6 days' white light in CIM followed by white light throughout SIM;

plants

24hR-W, early 24 hours red light shifting to 6 days' white light in CIM, followed by white light treatment in SIM; CIM, callus induction medium; SIM, shoot induction medium.

Table S1. The meristem development genes were found according to GO analysis

GO Term	GO Function	Gene
GO:0010492 GO:0030154	cell differentiation; maintenance of shoot apical meristem identity	*WUS*
GO:0019827 GO:0009934	stem cell population maintenance; regulation of meristem structural organization	*STM*
GO:0010072 GO:0010223	primary shoot apical meristem specification; secondary shoot formation	*CUC1*
GO:0090709; GO:0048366 GO:0010072	regulation of timing of plant organ formation; primary shoot apical meristem specification; leaf development	*CUC2*
GO:0009733 GO:1902459	response to auxin; positive regulation of stem cell population maintenance	*WOX5*
GO:1905392 GO:0010492	plant organ morphogenesis; maintenance of shoot apical meristem identity	*PLT3*
GO:0010311 GO:0045893	lateral root formation; positive regulation of transcription	*LBD16*
GO:0010089; GO:0045893 GO:0010311	xylem development; lateral root formation; positive regulation of transcription	*LBD18*
GO:0010262	somatic embryogenesis	*AGL15*

plants MDPI

Table S2. The genes of response to red light, far red light and dark were found according to GO analysis

GO Term	GO Function	Gene
GO:0010202, GO:0010203	response to low fluence red	AT2G18790
	light stimulus	AT1G09570
GO:0010114, GO:0009639	response to red light or far red	AT1G64860
	light	AT5G45340
GO:0009585, GO:0031516	red, far-red light	AT1G09530
GO:0031517	phototransduction	AT2G18790
GO:0055122	response to very low light	AT2G35720
GO:0009645	intensity stimulus	AT2G06850
GO:0009765, GO:0009768	photosynthesis, light	AT2G05100
GO:0009769	harvesting	AT3G11230
GO:0009646	response to absence of light	AT3G13450
GO:0009416	response to light stimulus	AT2G23050
GO:0071482	cellular response to light	AT5G13730
	stimulus	

Table S3. The genes of plant hormone response, transport, biosynthesis and oxygen signal were found according to GO analysis

GO Term	GO Function	Gene
GO:0009733	response to auxin	AT4G16950
GO:0010252	auxin homeostasis; auxin polar transport;	AT1G73590
GO:0009926	auxin efflux transmembrane transporter	AT1G23080
	activity	
GO:0009734	auxin-activated signaling pathway	AT3G62100
GO:0010279	indole-3-acetic acid amido synthetase activity	AT1G59500
GO:0009688	abscisic acid biosynthetic process; abscisic acid	AT4G18350
GO:0080168	transport	AT1G71960
GO:0009739	response to gibberellin; gibberellin	AT3G49850

GO:0009686	biosynthetic process	AT1G44090
GO:0009735	response to cytokinin; cytokinin-activated	AT3G47620
GO:0009736	signaling pathway	AT1G49190
GO:0009742	brassinosteroid mediated signaling pathway	AT1G19350
GO:0009753	response to jasmonic acid; regulation of	AT1G19180
GO:0009753	jasmonic acid mediated signaling pathway	AT1G06180
	oxidoreductase activity, acting on paired	AT3G48320
GO:0016709	donors, with incorporation or reduction of	AT1G58265
	molecular oxygen, NAD(P)Has one donor, and	AT5G42590
	incorporation of one atom of oxygen	
